# Brain plasticity following corpus callosum agenesis or loss: a review of the Probst bundles

**DOI:** 10.3389/fnana.2023.1296779

**Published:** 2023-11-06

**Authors:** Zorana Lynton, Rodrigo Suárez, Laura R. Fenlon

**Affiliations:** ^1^School of Biomedical Sciences, Faculty of Medicine, The University of Queensland, St Lucia, QLD, Australia; ^2^Queensland Brain Institute, The University of Queensland, St Lucia, QLD, Australia

**Keywords:** interhemispheric communication, brain development, callosotomy, commissurotomy, split brain, neurodevelopmental plasticity, ectopic tracts, longitudinal callosal bundle

## Abstract

The corpus callosum is the largest axonal tract in the human brain, connecting the left and right cortical hemipheres. This structure is affected in myriad human neurodevelopmental disorders, and can be entirely absent as a result of congenital or surgical causes. The age when callosal loss occurs, for example via surgical section in cases of refractory epilepsy, correlates with resulting brain morphology and neuropsychological outcomes, whereby an earlier loss generally produces relatively improved interhemispheric connectivity compared to a loss in adulthood (known as the “Sperry’s paradox”). However, the mechanisms behind these age-dependent differences remain unclear. Perhaps the best documented and most striking of the plastic changes that occur due to developmental, but not adult, callosal loss is the formation of large, bilateral, longitudinal ectopic tracts termed Probst bundles. Despite over 100 years of research into these ectopic tracts, which are the largest and best described stereotypical ectopic brain tracts in humans, much remains unclear about them. Here, we review the anatomy of the Probst bundles, along with evidence for their faciliatory or detrimental function, the required conditions for their formation, patterns of etiology, and mechanisms of development. We provide hypotheses for many of the remaining mysteries of the Probst bundles, including their possible relationship to preserved interhemispheric communication following corpus callosum absence. Future research into naturally occurring plastic tracts such as Probst bundles will help to inform the general rules governing axon plasticity and disorders of brain miswiring.

## 1. Introduction

The corpus callosum is the largest white matter tract connecting the right and left neocortical hemispheres and is exclusively present in eutherian (placental) mammals (Suárez et al., 2014, 2018). It is predominantly composed of axonal projections from cortical layers 2 and 3 (80%) and 5 (20%) pyramidal neurons, making both homotopic (symmetrical) and heterotopic (asymmetrical; 75%) connections (Fame et al., 2011; Fenlon and Richards, 2015; Szczupak et al., 2023a). Each cerebral hemisphere has lateralized functions, which are particularly pronounced in humans, and the corpus callosum is instrumental in integrating interhemispheric information for unified neuropsychological function. Pioneering work by Roger Sperry demonstrated that surgical ablation of the corpus callosum along the midline, termed “callosotomy,” can result in a disconnection, or “split-brain,” syndrome, characterized by mild to severe neuropsychological symptoms where lateralized functions fail to integrate (Sperry, 1961). In contrast, individuals with a developmental callosal absence, a condition called agenesis of the corpus callosum (ACC, which occurs approximately 1:4,000 live births, Guillem et al., 2003; Wang et al., 2004), as well as children who receive callosotomy early in life (before puberty), demonstrate some preserved interhemispheric connectivity as revealed by behavioral and resting-state functional magnetic resonance imaging studies (Jeeves, 1969; Lassonde et al., 1986, 1988, 1991; Paul et al., 2007; Jea et al., 2008; Tyszka et al., 2011; Owen et al., 2013; Roland et al., 2017). These distinct functional outcomes for different ages of callosal loss were first noted by Roger Sperry, and are known as the “Sperry paradox” (Sperry, 1968). The physical substrates that underlie this age-dependent plasticity remain unclear, but it has been suggested that compensatory rewiring through alternative routes may be involved (Tovar-Moll et al., 2014). This review focuses on the most prominent morphological feature present in the brains of many humans and animals with developmental, but not adult, loss of the corpus callosum: Probst bundles (PBs).

PBs, also known as longitudinal callosal bundles, are bilateral rostrocaudal fiber tracts that usually form in cases of agenesis of the corpus callosum (ACC) where the rest of the brain is not severely disorganized. Unlike other examples of developmentally aberrant axons that are eventually pruned, PBs are remarkably preserved into adulthood. The earliest description of these tracts was by Eichler in 1878 in a gross human specimen where it was noted that “the longitudinal ridge” exists as a “rudimentarily developed” corpus callosum (Eichler, 1878). It was later confirmed by the psychiatrist Moritz Probst that would-be callosal axons create this tract, which is not typically present in mammalian brains (Probst, 1901). Three decades later, homologous aberrant bundles were reported in an ACC mouse (King and Keeler, 1932), and mouse lines reliably resulting in spontaneous ACC were subsequently described (Wahlsten, 1974, 1982, 1987), allowing for this structure to be studied in more detail and experimentally manipulated. Since then, PBs have also been identified concomitant with ACC in hamsters (Lent, 1983), dogs (Wang-Leandro et al., 2018; Johnson et al., 2019), and rabbits (D’Arceuil et al., 2008). Given that there is phenotypic convergence of PBs arising from a variety of etiologies and across species, it is likely that there are conserved neuroplasticity mechanisms that reliably produce these ectopic and persistent tracts across eutherian mammals.

Despite being historically hypothesized to be tangled bundles of axons with little to no organization or function, here we review evidence suggesting that PBs are in fact stereotypically formed, organized, and may be functionally significant. Evidence from humans, mice, and hamsters gives insight into the potential faciliatory and/or detrimental functions of PBs, the required conditions for their formation, their various etiologies and developmental mechanisms. Detailed studies of naturally occurring stereotypical ectopic tracts such as the PBs may allow us to ultimately understand whether specific plastic anatomical changes are adaptive, maladaptive, or neutral, and whether therapies encouraging or inhibiting their formation in humans could be beneficial to functional outcome. Insights into PBs might therefore also extend to other injuries/developmental malformations of the brain and help us to understand the general rules governing axon plasticity and brain wiring disorders.

## 2. Probst bundle anatomy during development and at maturation

There is a vast diversity of etiologies underlying PB formation, including approximately 115 different gene mutations in mice and humans, as well as several cellular and mechanical influences (See section 3). While all PBs are broadly morphologically similar in their fiber orientation and gross position in the brain, an ongoing question in the field is whether all PBs have equivalent morphologies at a more detailed level, or instead whether they are diverse and categorizable (Gaudfernau et al., 2021). To better understand this, we compiled a systematic anatomical description of PB developmental and adult morphology.

### 2.1. Probst bundle development

The axons composing PBs originate from similar cortical projection neurons that form the corpus callosum in neurotypical brains. However, whether all neurons that would typically contribute to the corpus callosum also contribute to PBs remains unclear (Orioli et al., 1996; Demyanenko et al., 1999). A neurotypical human corpus callosum comprises axons from the frontal to occipital cortices, and follows a pattern of increased axon density rostrally, tapering through the mid-callosum, and an increased axon density caudally at its posterior pole, where axon density is correlated to cross-sectional area (Aboitiz et al., 1992). Like the corpus callosum, the PB also has a larger cross-sectional area rostrally, however it does not have a high density of axons caudally. Instead, PBs taper smoothly from rostral to caudal, with fibers most likely to originate from the frontal cortex, then the parietal cortex, and least likely from the occipital cortex, as revealed by histological tracing and DTI studies in rodents (Lent, 1984; Ozaki and Wahlsten, 1993; Ren et al., 2007) and MRI studies in humans (Meyer and Röricht, 1998). This raises the question of whether there are differences in the neurons along the rostro-caudal axis that contribute to the PB compared with the corpus callosum, or whether differences in neuronal cell death due to developmental pruning in brains with PBs may explain this phenomenon.

We reviewed studies of cell death that compared early callosotomy (conducted postnatally, within 1 day post birth in rodents, before most of the callosal axons cross the midline) with their genetically similar neurotypical controls. Rodent callosotomy experiments are particularly relevant for this comparison, as rodent and human congenital ACC cases are confounded by the possible contribution of involved genes also affecting cortical neuron number or density. One study histologically examined the differences in cortical thickness and neuronal density in multiple areas along the rostrocaudal axis in mice (Ribeiro-Carvalho et al., 2006), reporting a decrease in neuron number in areas of the cortex that would typically have large contributions to the corpus callosum. Conversely, a report in hamsters showed no differences in neuron number in the equivalent areas as measured in the previous study (Windrem et al., 1988). As these studies did not report a high-caudal to low-rostral gradient of cell death along the cortex, it is likely that additional mechanisms may contribute to the differing rostrocaudal axon densities observed between the corpus callosum and PBs. For example, PBs have increased axonal bifurcations in rostral areas (Rayêe et al., 2021), perhaps linked to the higher overall heterotopicity of projections recently reported in human and mouse brains with callosal malformations (Szczupak et al., 2023b) and contributing to differences in axonal bulk along the rostro-caudal axis.

**Figure 1:**
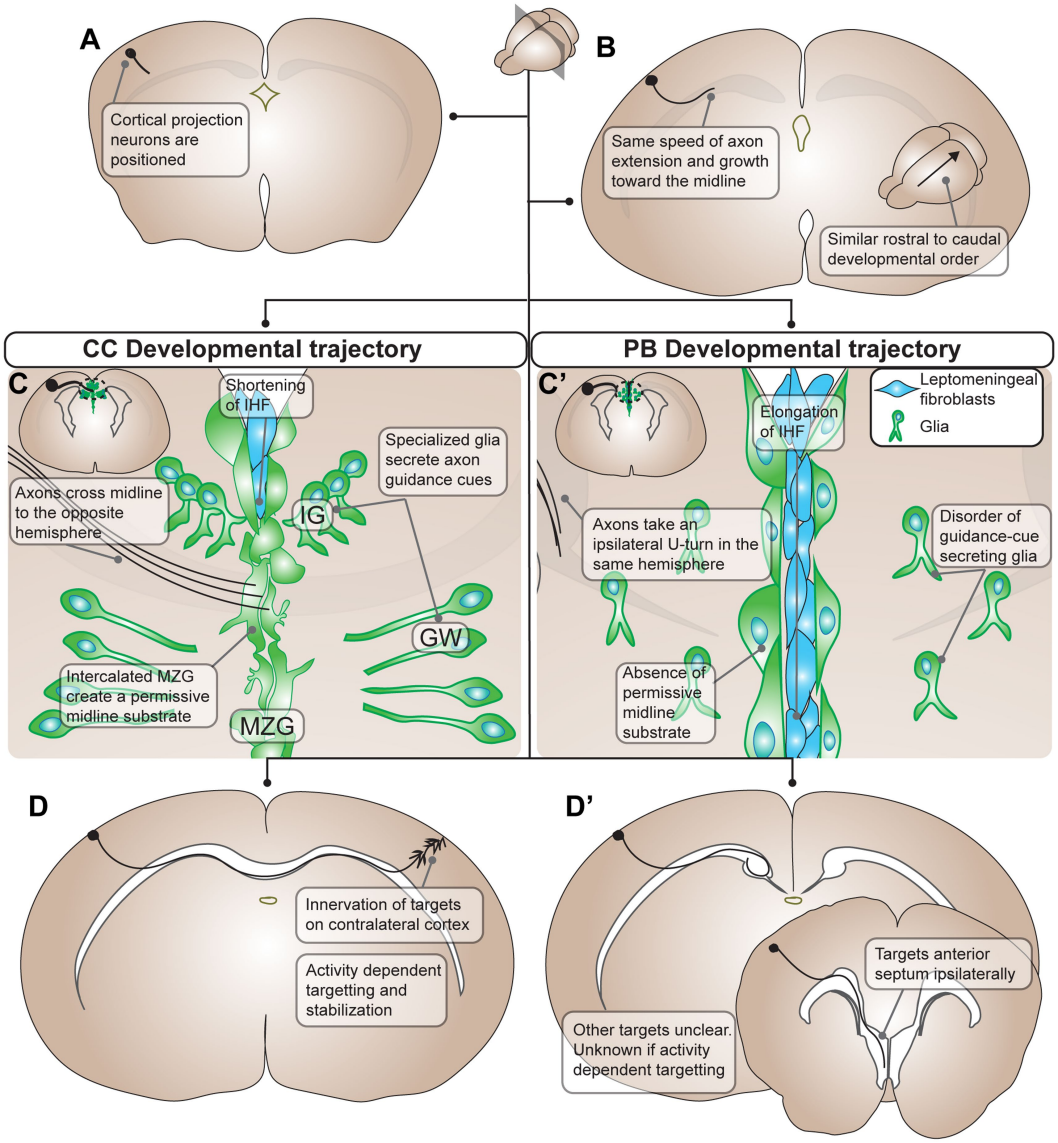
Summary of developmental processes of Probst Bundles (PBs) compared to the corpus callosum (CC) in mouse. **(A)** The development of the PB and CC starts out the same with differentiation and positioning of cortical projection neurons, though it is unclear if the exact same population of cortical projection neurons participates in the development of both the PB and the CC. **(B)** Cortical projection neurons extend axons medially toward the midline with the same speed of axon extension and growth in both PB and CC development. Additionally, there is a similar rostral to caudal developmental order in both the PB and the CC. **(C)** The developmental trajectories of the PB and CC diverges when axons contact the midline. In the CC, there is positioning of midline glia (green cells, in the indusium griseum, glial wedge, and midline zipper glia) that secrete axon guidance cues for the cortical projection axons. As well, midline zipper glia intercalate and remodel the midline, displacing leptomeningeal fibroblasts (blue cells) and subsequently shortening the interhemispheric fissure. This remodeling results in a permissive midline substrate for axons to cross. Axons of the CC respond to axon guidance cues from midline glia and penetrate the permissive midline substrate to cross to the opposite hemisphere. **(C’)** In PB development there is often disorder of guidance-cue secreting midline glia, for example through their mispositioning, malformation, or inability to secrete guidance cues at the ideal concentration. Additionally, midline zipper glia do not intercalate at the midline, resulting in subsequent elongation of the interhemispheric fissure and a midline substrate impermissive to axon extension. As a result, PB axons do not penetrate the midline and instead take an ipsilateral U-turn in the same hemisphere. **(D)** In the final stages of development, CC axons innervate targets on the contralateral cortex using activity dependent targeting and stabilization. **(D’)** PB axons target ipsilateral brain structures including the anterior septum ipsilaterally. Other targets of PBs are still unclear, and whether they use activity dependent mechanisms in targeting is unknown. PB, Probst bundle; CC, corpus callosum; IG, indusium griseum; GW, glial wedge; MZG, midline zipper glia.

Despite potential differences in the neurons that contribute to either the corpus callosum or the PB, the initial stages of development of these tracts are remarkably similar (Figure 1). At the earliest stages of development, neural tracing studies with the lipophilic carbocyanine dyes DiI/DiA in mice reported no differences in the first steps of PB and callosal development, including similar speeds of axon extension and growth toward the midline, as well as a similar rostral to caudal developmental order in both the corpus callosum and PB (Ozaki and Wahlsten, 1993). The presumptive PB starts to morphologically diverge from a neurotypical corpus callosum only at the equivalent developmental stage at which callosal axons begin to traverse the midline (Silver et al., 1982; Wahlsten et al., 2006). The callosal axons contact and cross the midline through a glia-rich permissive midline substrate and respond to axon guidance cues secreted in the area to cross to the contralateral cortex (Figure 1). The formation of the midline substrate is dependent upon appropriate remodeling of the interhemispheric fissure at the cortico-septal midline, and the absence of a remodeled midline is causally related to ACC, an elongated interhemispheric fissure, and subsequent PB formation (Gobius et al., 2016). The PB axons, in contrast to corpus callosum axons, make a sharp longitudinal turn as they approach the midline, likely following cues that include disrupted midline guide-post cells, spatial and physical constraints, axon contacts with a retained/unmodelled interhemispheric fissure, altered axon guidance molecules, and/or additional mechanisms (detailed in Section 3). It has also been reported that callosal axons transiently bifurcate before reaching their midline targets, and that these bifurcations occur differentially depending on where in the cortex the neurons are located (Garcez et al., 2007). An intriguing hypothesis is that tendency to bifurcate prior to midline crossing might encourage some axons to take alternative routes, such as form a PB. Future studies comparing cortical areas likely to contribute to PBs in ACC brains with areas most predisposed to bifurcation may help to clarify whether these phenomena are related.

After this divergence in axon trajectory, the corpus callosum and PB resume many of their shared developmental characteristics. Both form fasciculated axon tracts (Wahlsten et al., 2006), and a DTI study in fetal humans reported that both have similar fractional anisotropy values at different developmental stages (Kasprian et al., 2013), suggesting that some callosal axon guidance mechanisms and maturation programs may be retained by PBs. Indeed, the developmental mechanisms underlying callosal formation (as well as the growth of other brain connections) can be used to provide clues about the development and functionality of the PB. For example, in many circumstances, callosal axons projecting to incorrect or inappropriate sites are eliminated during neurodevelopment (Yamaguchi and Miura, 2015; Yamaguchi et al., 2018). Intriguingly, despite constituting very large ectopic tracts, PBs largely avoid elimination and persist into adulthood, suggesting that they may be making functional synaptic connections during development, which are perhaps retained into adulthood (see Section 4). The corpus callosum also specifically uses electrical activity as a cue to guide axon targeting (Huang et al., 2013; Suarez et al., 2014), and it has been further shown that spatially symmetrical bilateral activity is necessary for normal contralateral callosal targeting (Suarez et al., 2014; De León Reyes et al., 2019; Babij et al., 2023). Whether these principles of neurodevelopment are also used by the PBs to inform patterns of connectivity remains largely unexplored, but more in-depth studies could help to clarify the mechanisms underlying PB formation and their final structure and function.

### 2.2. Probst bundle anatomy

**Figure 2:**
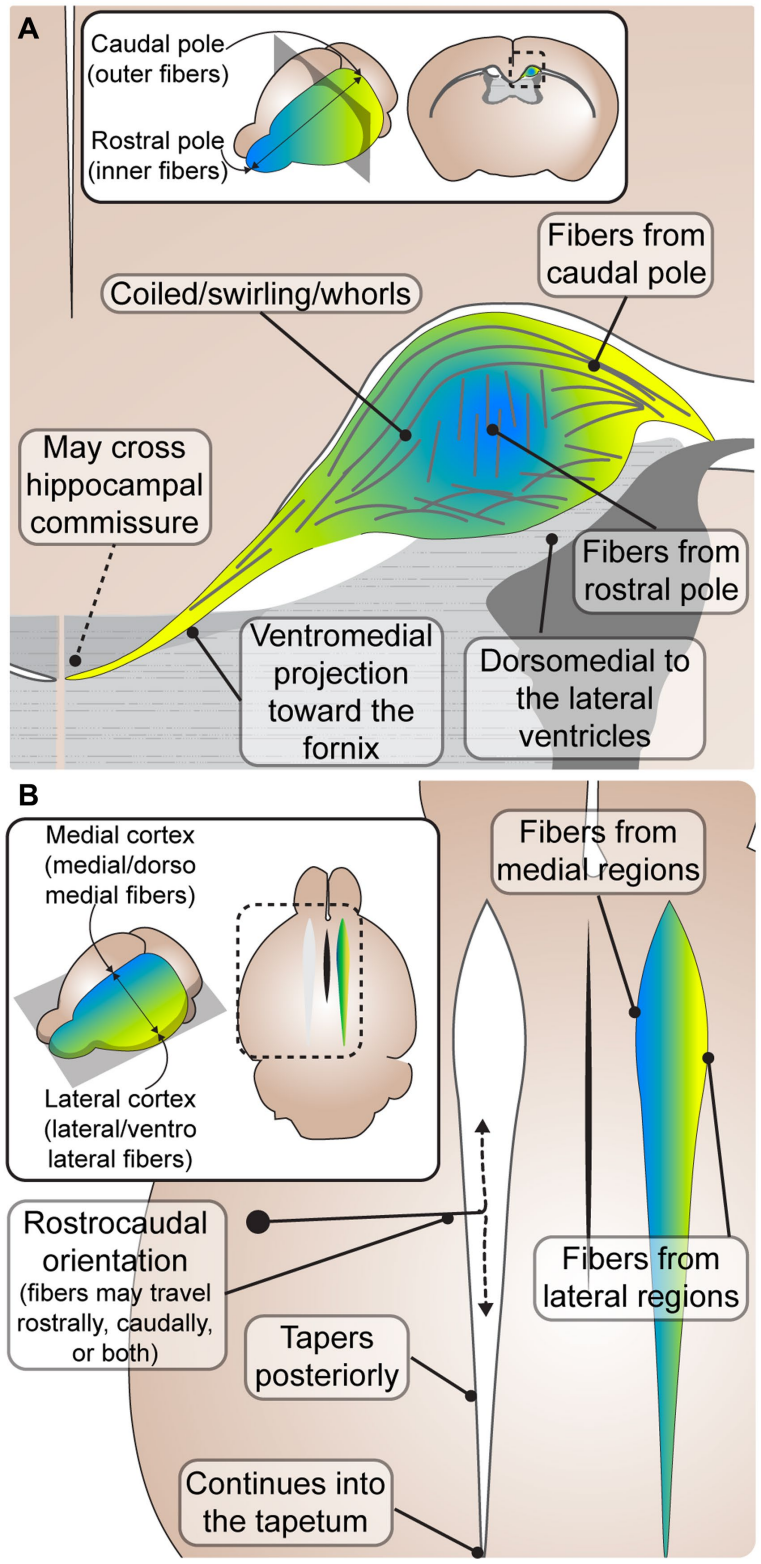
Common characteristics of Probst bundles (PBs) in mouse and human. **(A)** A coronal schematic depicting an adult PB in mouse. PBs are consistently described as fibers that coil in a close dorsomedial relationship to the lateral ventricles. Some fibers leave the PB in a ventromedial projection toward the fornix, where it has been reported that some fibers may cross the hippocampal commissure to the contralateral hemisphere. Inset depicts the rostral (blue) and caudal (yellow) orientation of fibers, which are topographically represented in the close-up schematic with inner fibers from the rostral pole and outer fibers from the caudal pole. **(B)** A horizontal schematic depicting the rostrocaudal orientation of PB fibers, where fibers may travel from rostral to caudal, caudal to rostral, or both. The PB tapers posteriorly, continuing into the tapetum. Inset depicts the medial (blue) and lateral (yellow) orientation of fibers, which are topographically represented in the close-up schematic with medial fibers from medial brain regions, and lateral fibers from lateral brain regions.

To gain insight into the relative homology or heterogeneity of PB microanatomy, we analyzed articles describing detailed PB anatomy in mammals with complete ACC and quantified the frequency with which common features were reported (Table 1 and Supplementary Table S1). A total of 28 reports were identified with descriptions of PB anatomy at various stages of development. No articles explicitly contradicted any of the anatomical features listed in Table 1. The features identified, from most to least commonly reported, include a rostro-caudal fiber orientation, a dorsomedial relationship with the lateral ventricle, a coiled morphology, a ventromedial projection to the fornix, topographic organization, decreasing diameter along the rostro-caudal axis, and a continuation posteriorly into the tapetum of the lateral ventricles. All of these features were also identified in the specimen examined by Probst in the original paper from 1901 (Probst, 1901). These anatomical features are discussed in greater detail in the following sections, and are summarized in Figure 2.

**Table 1:** Summary of anatomical PB features in complete ACC described in mouse, human, and hamster.

Anatomy	Number of papers reporting presence of feature/Number of papers reporting absence of feature	Citations
Dorsomedial relationship with LV	11/0	2, 3, 4, 6, 9, 10, 14, 15, 16, 17, 28
Coiled/swirling/whorls	8/0	4, 5, 7, 12, 20, 21, 22, 24
VM Projection to fornix	7/0	7, 11, 12, 14, 20, 21, 28
Rostro-caudal fiber orientation	14/0	2, 3, 4, 5, 6, 7, 10, 11, 13, 17, 18, 20, 22, 24
Decreasing diameter along the rostro-caudal axis	5/0	4, 11, 15, 18, 21
Continues posteriorly into the tapetum	4/0	4, 9, 13, 15
Topographically organized	5/0	11, 18, 20, 26, 27

A total of 28 reports were identified with descriptions of PB anatomy at various stages of development [unknown (1), embryonic (5), early postnatal (11), and adult (19)], and various mammals [mouse (12), hamster (2), and human (14)]. Articles describing detailed PB anatomy were selected following a search on PubMed and Google Scholar for articles that included “Probst Bundle” or “longitudinal callosal bundle” in the text. Articles were selected if they included PB descriptions in complete ACC and were written in English. Citation key: [2] (Barkovich and Norman, 1988), [3] (Bénézit et al., 2015), [4] (De Lange, 1925), [5] (Dodero et al., 2013), [6] (Kasprian et al., 2013), [7] (King, 1936), [9] (Kirschbaum, 1947), [10] (Lee et al., 2004), [11] (Lent, 1984), [12] (Lent, 1983), [13] (Loeser and Alvord, 1968), [14] (Magee and Olson, 1961), [15] (Meyer and Röricht, 1998), [16] (Meyer et al., 1998), [17] (Mitter et al., 2015), [18] (Ozaki et al., 1989), [20] (Ozaki and Wahlsten, 1993), [21] (Ozaki et al., 1987), [22] (Ren et al., 2007), [24] (Silver et al., 1982), [26] (Tovar-Moll et al., 2007), [27] (Utsunomiya et al., 2006), [28] (Yousefi and Kokhaei, 2009).

The most common feature of PB anatomy reported was its orientation along the rostrocaudal axis (King, 1936; Barkovich and Norman, 1988; Ozaki et al., 1989; Meixner et al., 2000; Lee et al., 2004; Dodero et al., 2013; Kasprian et al., 2013; Mitter et al., 2015). However, the exact directionality (e.g., caudal cell bodies sending axon projections rostrally, caudally, or both) remains less clear. PBs are frequently described to be oriented from rostral to caudal, or running in the rostrocaudal direction (De Lange, 1925; Loeser and Alvord, 1968; Silver et al., 1982; Lent, 1984; Ren et al., 2007; Bénézit et al., 2015). However, studies describing the development of PBs with DiI tracing across different early developmental stages revealed that axons stretch longitudinally in both a rostral and caudal direction (Ozaki and Wahlsten, 1993), and a later histological tract tracing study in adult mice reported that axons project from the caudal somatosensory cortex to more rostral motor areas (Chen et al., 2005). It is possible that the longitudinal directionality of PB is mixed in earlier developmental stages, and later prunes into a primarily rostral or caudal trajectory in different regions, however more precise tract-tracing studies across ontogeny are required to elucidate this.

PBs are also frequently reported to run dorsally and medially to the lateral ventricles (De Lange, 1925; Kirschbaum, 1947; Magee and Olson, 1961; Barkovich and Norman, 1988; Meyer et al., 1998; Meyer and Röricht, 1998; Lee et al., 2004; Yousefi and Kokhaei, 2009; Kasprian et al., 2013; Bénézit et al., 2015; Mitter et al., 2015), and extend into the caudal tapetum (De Lange, 1925; Kirschbaum, 1947; Loeser and Alvord, 1968; Meyer and Röricht, 1998). The PBs have been hypothesized to cause the morphological indenting of the rostral lateral ventricles in humans, with dilation of the caudal portions, resulting in colpocephaly (Kirschbaum, 1947; Meyer et al., 1998; Meyer and Röricht, 1998; Bénézit et al., 2015). Indeed, the rostro-caudal tapering of the PB is directly correlated with the caudo-rostral tapering of the lateral ventricles, and a study reported a high association between the presence of PBs and colpocephaly in human ACC (Al-Hashim et al., 2016). However, whether PB formation causally leads to colpocephaly requires further study.

PBs are also frequently described as having a tortuous configuration (De Lange, 1925; King, 1936; Silver et al., 1982; Lent, 1983; Smith et al., 1986; Ozaki et al., 1987; Ozaki and Wahlsten, 1993; Wahlsten et al., 2006; Ren et al., 2007; Dodero et al., 2013), and a disorganized structure (King, 1936; Lent, 1984; Ozaki et al., 1987; Ozaki and Wahlsten, 1993; Ren et al., 2007). However, within this seemingly disorganized structure, a consistent topographic arrangement has been described (Lent, 1984; Ozaki and Shimada, 1988; Ozaki et al., 1989; Ozaki and Wahlsten, 1993; Utsunomiya et al., 2006; Tovar-Moll et al., 2007). Fibers from the medial cortex course medially (Lent, 1984) and/or dorsomedially (Ozaki and Wahlsten, 1993) within the bundles, while fibers extending from the lateral cortex course laterally (Lent, 1984) and/or ventrolaterally (Ozaki and Wahlsten, 1993; Figure 2B). Diffusion tensor imaging studies corroborate histological data, supporting a topographic arrangement within the PB (Tovar-Moll et al., 2007), with fibers from the frontal pole running on the innermost side of the PB and fibers from the orbital gyri running along the outermost side (Utsunomiya et al., 2006; Figure 2). The lack of contradiction of these features in any of the analyzed articles suggests that PBs likely have a relatively conserved gross anatomy among diverse species and etiologies. The topographic arrangement within PBs with respect to cells of origin may be related to a similar organization of fibers within the corpus callosum, where more rostral areas are located more rostrally within the tract, and more dorsal areas located more dorsally (de Lacoste et al., 1985; Tovar-Moll et al., 2007; Zhou et al., 2013). Interestingly, this arrangement is also present in the neocortical connections coursing through the anterior commissure in marsupials and monotremes that do not have a corpus callosum (Suárez et al., 2018), suggesting that cortical axon topography within the white matter is an ancient feature of intercortical connectivity. The arrangement of axons within the corpus callosum is known to regulate patterns of contralateral homotopic connectivity (Zhou et al., 2013) and may be related to the known rostral-caudal and lateral-medial sequential order of cell birth and maturation in the neocortex. Recent findings reporting that around 75% of callosal connections are heterotopic in mice, marmosets and humans suggest that the developmental cues guiding callosal axons may be more multidirectional than previously thought, and may offer insight into potential mechanisms of their formation (Szczupak et al., 2023a). Future studies investigating these relationships in both the neurotypical corpus callosum and PBs may help to uncover the mechanisms driving topographic arrangement, as well as the temporal order and regional specificity of PB formation.

Precisely how features of Probst bundles might differ across a variety of etiologies and/or species is poorly understood, and therefore what constitutes a PB may vary based on interpretation. There are reports of “PB-like” fibers, or labeling of aberrant bundles, that do not fit the classical morphological description described in the section above (Supplementary Table S2). These unusual PB cases did not fit our criteria, were exceedingly rare, or it could be argued that they are not true PBs as were defined originally by Probst (1901). In some cases, PBs were described but axons appeared halted at the midline in either a case of arrested growth, or with the formation of aberrant neuromas directly abutting the midline (Supplementary Table S2). Many of these reports included prenatal brain specimens in which it may be too early to assess whether stalled axonal growth would have ultimately formed PBs, a neurotypical corpus callosum or another phenotype. In some cases, different mouse models with the same gene manipulated (e.g., a cortex-specific conditional knockout, where the complete knockout was perinatally lethal) were reported to result in obvious PB morphology at a later developmental stage, in which case these later reports were included in subsequent analyses. In other cases, there were contradictory reports as to whether a particular model results in the development of PBs, perhaps due to subtle genetic differences of mouse lines/backgrounds or differences in experimental protocols. Thus, our criteria for inclusion of PBs in the following sections was based on the presence of anatomical features shown in Figure 2 where histological images were able to be reviewed. Where histological images were not shown, the phenotype was categorized as described by the study.

### 2.3. Probst bundle projections

Several reports indicate that fibers contributing to the PBs do not remain within the fasciculated tract, but rather project out into various brain locations. In humans, these patterns of connectivity have been reported to be relatively consistent between subjects (Owen et al., 2013), however the precise PB connectome and its degree of variability in both rodents and humans remains unclear. Histological studies in rodents have reported that PBs project broadly to the same ipsilateral areas innervated by the corpus callosum (Ozaki et al., 1989), and DTI studies in humans (Bénézit et al., 2015) have similarly reported a broad ipsilateral PB projection pattern in keeping with that of a neurotypical corpus callosum projecting contralaterally, including into the frontal, parietal, occipital and temporal lobes. However, reported differences in PB projections to the cortex include a more exuberant ipsilateral projection pattern than the ipsilateral projection pattern of similarly labeled neurons in neurotypical brains (Ozaki et al., 1989), particularly to more rostral regions of the brain such as the frontal lobes (Tovar-Moll et al., 2007; Kasprian et al., 2013), as well as potentially a bias to more paramedial than lateral cortical regions (Owen et al., 2013). This rostral exuberance, as well as the rostrocaudal axis of PBs, may contribute to the reported increase in rostrocaudal connectivity in human subjects with ACC (Tovar-Moll et al., 2007; Owen et al., 2013; Bénézit et al., 2015; Jakab et al., 2015). Some of the anatomical features of PBs resemble those of the neurotypical cingulate bundle, or cingulum, which runs rostrocaudally over the corpus callosum to connect ipsilateral cortical hubs along the midline (regions that are also heavily connected interhemispherically by the corpus callosum), as well as extracortical regions that include the thalamus, basal forebrain, hippocampal formation and other limbic regions (Bubb et al., 2018).

**Figure 3:**
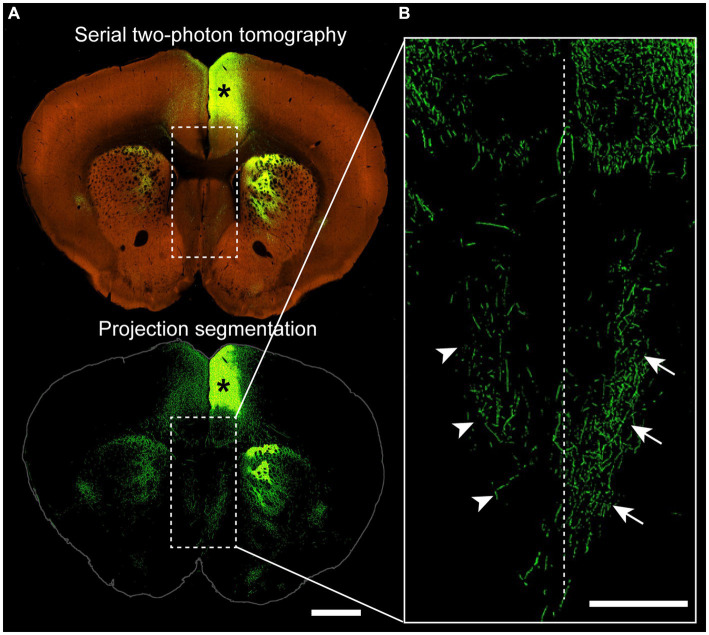
Projections from the cingulate cortex to the ipsilateral and contralateral septum. **(A)** Coronal section of a wildtype mouse (C57BL/6 J) injected with adeno-associated virus encoding green fluorescent protein into the anterior cingulate cortex (asterisk), with a two-photon tomography (top) and a projection segmentation view (bottom) highlighting the distribution of axon terminals. **(B)** Higher magnification image from the corticoseptal region outlined in **(A)** demonstrating ipsilateral (arrows) and contralateral (arrowheads) projections. Dotted line indicates the interhemispheric midline. Allen Mouse Brain Connectivity Atlas, https://connectivity.brain-map.org/projection/experiment/cortical_map/146593590. Scale bars: 1,000 μm in **(A)**, 500 μm in **(B)**.

Another exuberant/ectopic projection found in ACC brains that may contribute to this rostral exuberance is a dense projection that courses ventrally from the PB into the ipsilateral anterior septum (Ozaki and Wahlsten, 1993; Gu et al., 2003; Chen et al., 2005; López-Bendito et al., 2007; Islam et al., 2009; Niquille et al., 2009; Piper et al., 2009; Bilasy et al., 2011; Conway et al., 2011; Benadiba et al., 2012; Fothergill et al., 2014; Laclef et al., 2015), which is even evident in studies that do not explicitly report it (Silver et al., 1982; Shu et al., 2003; Islam et al., 2009), with cell bodies of origin hypothesized to be located in the cingulate cortex, which is the location of the cell bodies that first extend axons to pioneer the corpus callosum in neurotypical individuals (Piper et al., 2009). Accordingly, anterograde tracer injections into the cingulate cortex of neurotypical mice reveal projections to the anterior septum of both ipsi- and contralateral hemispheres, further suggesting that PBs might exploit at least some of cingulum bundle targeting mechanism (Figure 3). Several neural tracing studies in ACC animals identified exuberant axons in the anterior septum arising from cortical areas that usually do not project to these regions, suggesting that PBs may be ectopically contributing to these established circuits. An ectopic septal projection was produced in more caudal regions of the brain due to misplaced glutamatergic neurons and Sema3C cells, providing a potential mechanism for these more anterior septal projections that arise from the PBs (Niquille et al., 2009). Ectopic projections to the septum have also been reported in ACC brains without PBs (Bagri et al., 2002; Andrews et al., 2006; Chinn, 2011; Conway et al., 2011; Unni et al., 2012), suggesting that the two structures are not necessarily always linked.

Given the remarkable ability of some patients with ACC to perform tasks that require bilateral integration, the possibility that PBs could contribute to other interhemispheric connections has been an intriguing topic for decades (further discussed in Section 4). Some fibers from the PB project ventromedially, with reports that they may join the fornix (King, 1936; Magee and Olson, 1961; Lent, 1983, 1984; Ozaki et al., 1987; Ozaki and Wahlsten, 1993; Yousefi and Kokhaei, 2009). Whether or not fibers from the PB contribute to the fornix and/or cross to the contralateral hemisphere over the hippocampal commissure is less clear. Of the 28 articles that met our criteria for morphological description (described in section 2.2), nine commented explicitly on the presence or absence of PB crossing at the midline, and an additional five reports were identified outside of those original 28 articles that commented on PB midline crossing. From this total of 14 PB midline crossing reports, all of which used histological tract tracing techniques, 12 stated that the PBs cross at the level of the hippocampal commissure (Silver et al., 1982; Lent, 1983, 1984; Ozaki et al., 1984, 1987, 1989; Olavarria et al., 1988; Ozaki and Wahlsten, 1993; Orioli et al., 1996; Qiu et al., 1996; Lanier et al., 1999; Molyneaux et al., 2005) and two reported that the PBs remain exclusively ipsilateral (King, 1936, Ren et al., 2007; Supplementary Table S3). The tract tracing methods used, however, do not have the precision to conclusively discern whether fibers within the hippocampal commissure originate from the PBs. Therefore, it remains unclear whether the hippocampal commissure is a common place for bilateral PBs to communicate with each other, and whether conflicting reports are reflective of bona fide PB interindividual variability, misidentified callosal remnants, or fibers from other sources that cross the hippocampal commissure in close proximity to the PBs.

In addition to reports on connectivity within cortical regions, there have also been histological reports of PBs projecting to subcortical targets in hamster (Lent, 1983). Although this has not been anatomically corroborated in humans, a virtual Probstostomy revealed an impact on connections between cortical and subcortical regions, suggesting that such connections, whether monosynaptic or polysynaptic, may be possible (Owen et al., 2013). Further work combining precise histology and DTI in different species and across different developmental etiologies of ACC will help to reach a consensus on the characterization of variability of PB projections, and ultimately help to predict and inform their potential for functional connectivity.

### 2.4. Probst bundle anatomy in partial agenesis of the corpus callosum

**Figure 4:**
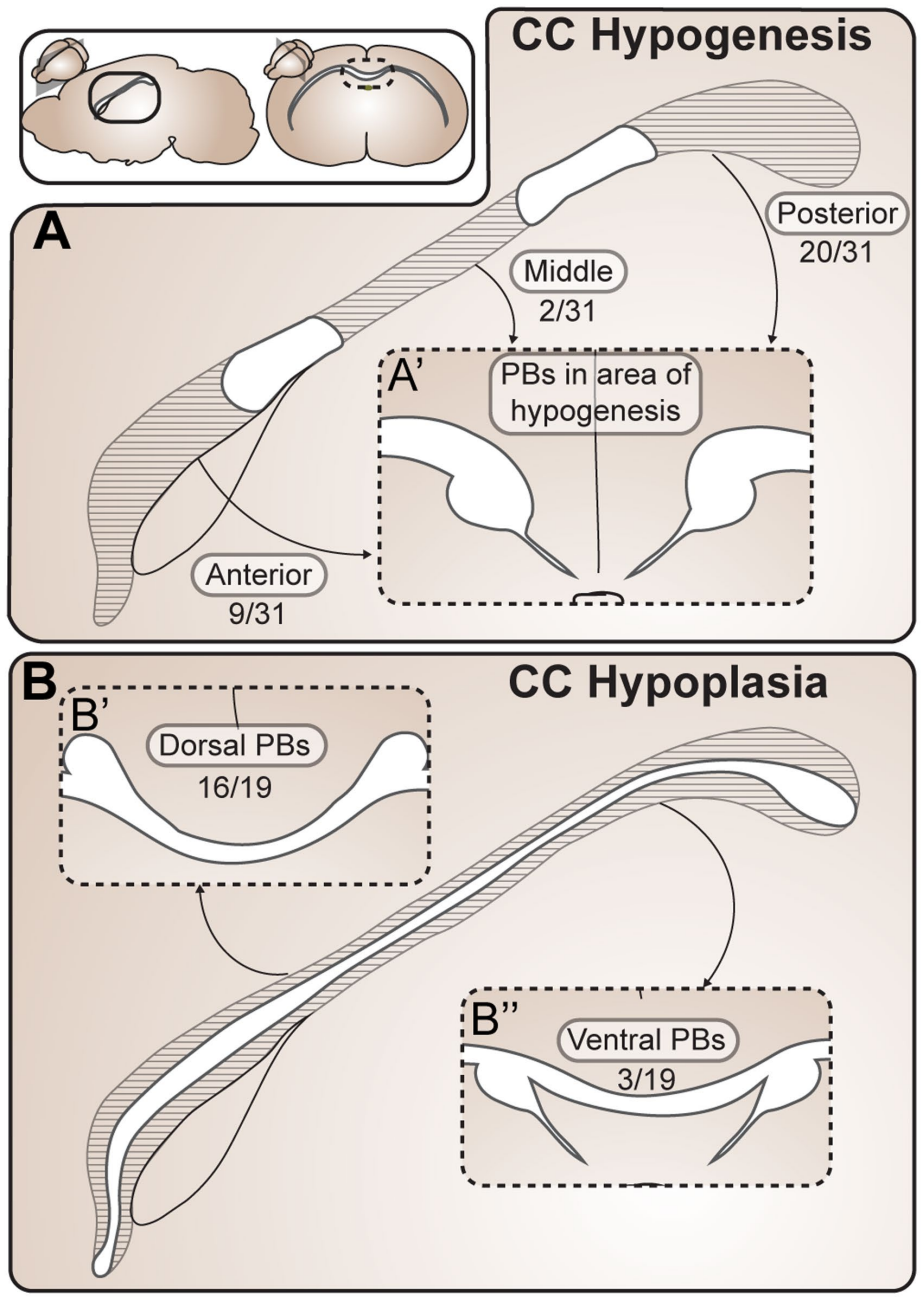
Probst Bundles (PBs) form in a variety of partial ACC phenotypes. **(A)** In CC partial hypogenesis there is agenesis of the CC in anterior, middle, and/or posterior portions along its rostrocaudal axis. Reports of PB formation in CC partial hypogenesis in mammals were grouped according to location of PB formation in relationship to the callosal remnant, including 31 cases of CC hypogenesis **(A)** and 19 cases of CC hypoplasia **(B)**. PBs have been reported to occur in all areas of partial hypogenesis **(A)**, with posterior PBs being the most frequent (20/31 cases), anterior PBs being less frequent (9/31 cases), and PBs forming in the mid-callosum being the least frequent (2/31 cases). In CC hypoplasia **(B)** there is thinning of the CC along its dorsocaudal axis, which can include dorsal and/or ventral CC fibers. Cases of PB formation in partial ACC hypoplasia in mammals were grouped according to location of PB formation in relationship to the thin callosum. PBs have been reported to occur more frequently dorsal to (16/19 cases) rather than ventral to (3/19 cases) the hypoplastic CC. ACC agenesis of the corpus callosum; CC, corpus callosum; PB Probst bundle.

PBs also form in brains where the corpus callosum is only partially absent, termed partial ACC (Figure 4). Partial ACC can be defined by having an ACC phenotype with a callosal remnant (a partial absence of the corpus callosum along its rostro-caudal axis: “partial hypogenesis”), or a hypoplastic corpus callosum (a thinning of the corpus callosum along its dorso-ventral axis). We reviewed reports of PBs that form in partial ACC and systematically categorized the locations of PB fibers in relationship to the corpus callosum based on images and/or descriptions. We identified 113 reports of partial ACC occurring with PBs in mice, dogs, rabbits, and humans, 43 of which provided adequate information for categorization (Supplementary Table S4). Ambiguous reports where it was unclear whether there was a very small callosal remnant in a small area above the hippocampal commissure, or whether PBs were connecting via the dorsal hippocampal commissure, were not included and are discussed elsewhere (Subsection 2.3).

Within the 43 reports, there were 19 cases of corpus callosum hypoplasia, 31 cases of corpus callosum partial hypogenesis, and one case that could not be placed into either category. Of the 19 cases of corpus callosum hypoplasia, PBs have been reported to occur dorsal to (16/19) and ventral to (3/19) a thin callosum. In the 31 cases of corpus callosum partial hypogenesis, PBs occurred posterior to (20/31), anterior to (9/31), and in the middle (2/31) of callosal remnants. This can be compared with an estimated frequency of anterior versus posterior remnants (with or without PBs) with a 92.5% frequency of anterior remnants versus 7.5% posterior remnants (Al-Hashim et al., 2016). If PBs were equally likely to form posterior or anterior to a callosal remnant, we would expect the cases of anterior and posterior PBs to be the same as the frequency of posterior and anterior callosal remnants. However, the percentage of PBs we identified forming posterior to the remnant (20 out of 31 cases, 65%) is lower than the estimated frequency of anterior (as opposed to posterior) remnants (as identified in Al-Hashim et al., 92.5%), indicating that PBs may be more likely to form in cases of anterior partial hypogenesis with posterior remnants. This is in keeping with a prior report of PB characterization in partial corpus callosum hypogenesis in humans identifying a high association between the presence of PBs and agenesis of the anterior callosal fibers, with PBs forming in only 3% of brains with an intact rostral callosum (Al-Hashim et al., 2016). Further investigation into the relative incidences of callosal remnant and PB location across species and etiologies will help to inform the developmental constraints of PB formation. For example, the variety of PB locations in cases of partial PB and the known rostral-to-caudal order of midline crossing during neurotypical callosal development (Richards et al., 2004) suggest that PBs may be able to form either before or after the successful crossing of part of a callosal tract, but may be more likely to form before successful crossing (in more anterior regions).

An additional intriguing case of an ectopic tract has been reported in some cases of partial corpus callosum hypogenesis: sigmoid bundles. Sigmoid bundles are aberrant fiber tracts that asymmetrically connect the frontal lobe to the contralateral parieto-occipital cortex via the callosal remnant and have been predicted via diffusion imaging in humans (Tovar-Moll et al., 2007; Wahl et al., 2009; Bénézit et al., 2015; Jakab et al., 2015) and histological tract-tracing in mouse models of partial corpus callosum hypogenesis (Edwards et al., 2020; Szczupak et al., 2020). An augmented coherence of EEG signal between the regions predicted to be interconnected by sigmoid bundles in partial corpus callosum hypogenesis subjects compared to neurotypical individuals further suggests that this tract may contribute to functional connectivity (Lazarev et al., 2016). It remains unclear to what extent partial PBs may contribute to the sigmoid bundle, or whether it forms as an independent tract and/or via independent mechanisms.

### 2.5. Possible mechanisms underlying Probst bundle anatomy

While the anatomy of PBs has been described by several researchers, the developmental mechanisms that govern their stereotypical route remain unknown. There are two predominant hypotheses that seek to explain PB anatomy: first, that PB morphology arises due to mispositioning of midline glial structures, and second, that they form by hijacking an existing association tract.

In neurotypical animals, callosal axon guidance depends on the positioning and guidance cues from specialized glia-rich guideposts at the midline: the indusium griseum, the glial wedge, and the midline zipper glia (Figure 1). In ACC with PB, these structures are frequently mispositioned, and thus it is hypothesized that their normally expressed guidance cues in aberrant locations drives PB directionality by attracting and repelling axons in different directions. Midline glia have been frequently reported to occur within and surrounding the PB structure itself, indicating that they may be providing guidance signals to the axons within (Silver et al., 1982; Mendes et al., 2006; Bilasy et al., 2011). However, there are also instances in which mouse models with ACC display grossly malformed midline glia populations, yet still form PBs with characteristic morphology (Islam et al., 2009; Rayêe et al., 2021) indicating that alternative or additional mechanisms may guide their development.

Alternatively, PBs might hijack axon guidance systems of other fiber tracts in neighboring regions that are spared in ACC and are also present in neurotypical brains. One possibility is that this could be facilitated by axon guidance ligands in the existing association tract that are complementary to receptors on the would-be callosal axons, ultimately encouraging axon growth and guidance along the alternative paths. It has also been reported in other systems that, in some cases, pioneering populations of axon tracts rely on guidance signals, while these signals are less necessary for follower axons (Raper and Mason, 2010). Follower axons may therefore be able to indiscriminately follow tracts that are already pioneered (e.g., via axo-axonal contacts and/or fasciculation) without the need for complementary guidance cues in the surrounding milieu. However, at present there is no clear evidence for this growth/guidance mechanism in the neurotypical corpus callosum, nor in PBs.

Perhaps the primary candidate of a neurotypical tract that might provide a physical scaffold and/or molecular cues used by PBs is the cingulum bundle. Cingulum bundles are longitudinal, bilateral tracts that interconnect diverse areas, including prelimbic, anterior cingulate, retrosplenial and occipital cortex bidirectionally, as well as extracortical areas such as the thalamus, brainstem and hippocampus (Bubb et al., 2018). Cingulum bundles are also present in ACC brains distinct from and dorsomedial to the PBs (Bénézit et al., 2015), demonstrating that PBs do not simply constitute an enlarged ectopic cingulum. However, their close apposition, similar directionality, and conservation of targets (e.g., varied cortical areas) provide an intriguing possibility of shared developmental mechanisms. Other tracts that run longitudinally and may provide potential candidates include the inferior longitudinal fasciculus, interior fronto-occipital fasciculus and superior fronto-occipital fasciculus projection, the latter of which has been described to connect the frontal and occipital lobes in monkeys (Forkel et al., 2014), but has not been definitively evidenced to exist in healthy adult humans. An expanded understanding of the developmental mechanisms of PBs as well as additional candidate neurotypical scaffold tracts will help to inform our understanding of their developmental relationships and dependencies.

## 3. The variety of genetic and structural etiologies underlying Probst bundle formation

As described in the section above, there is consistency in the gross anatomical features of PBs regardless of their structural or genetic cause. To better understand the diversity of etiologies that can lead to PB formation, as well as whether there are trends in those conditions that may point to the mechanisms underlying PB formation, we reviewed their potential genetic, structural, and environmental causes. Studies that specifically mentioned PBs and their likely etiologies in humans, mice, and hamsters were identified and the likely genetic, structural, or environmental etiology was recorded and categorized (Supplementary Table S5). We identified 231 reports of PBs with likely etiologies in mouse (189 cases, 157 with PBs, 32 without PBs), humans (127 cases, 90 with PBs 37 without PBs), and hamsters (3 cases with PBs) (Table 2).

**Table 2:** Etiologies of brains with ACC with or without PBs in mouse, hamster, and human.

Etiology	Number of cases identified out of 231 reports of ACC					
	Human		Mouse		Hamster	
PBs present	Yes	No	Yes	No	Yes	No
Structural	11	1	6	0	3	0
Genetic	75	33	149	31	0	0
Environmental	4	3	2	1	0	0
Total	90	37	157	32	3	0
	127		189		3	

There were 231 reports of ACC specifying the presence or absence of PBs with likely etiologies identified in mouse (189 cases), humans (127 cases), and hamsters (3 cases). The most common etiology reported in different cases was genetic.

### 3.1. Structural etiologies of Probst bundles

Structural disruption to the midline during development from a variety of causes, whether surgical or secondary to mass effect of a tumor or cyst, can lead to the development of PBs (Table 3). This has been most clearly demonstrated in rodent studies, where early surgical callosotomy conducted either embryonically as early as embryonic day (E)16 in mouse, or at early postnatal stages [postnatal day (P)0–1], consistently produces PBs in animals with a wildtype genetic background. Specifically, surgical lesioning of the glial populations that contribute to remodeling of the midline has been hypothesized to be the primary etiology of PB formation in these cases (Silver et al., 1982). This is further supported by reports of a partial rescue of the PB phenotype after surgical implantation of glial-coated implants at the midline in mice (Silver and Ogawa, 1983). However, as it is difficult to mechanically disrupt one small structure in isolation, the specific extent and nature of surgical disruption required to produce PBs remains unclear.

**Table 3:** Number of structural cases of ACC with PBs in humans, mice, and hamster, organized by type of defect.

Type of structural defect	Number of cases		Citations
	Rodent	Human	
Interhemispheric cyst	0	7	1, 9, 10, 11, 14, 15, 19
Lipoma	0	3	1, 13,18
Hamartoma	0	1	12
Early surgical midline lesioning	9	0	2, 3, 4, 5, 6, 7, 8, 16, 17

Citation key: [1] (Sztriha, 2005), [2] (Lent, 1984), [3] (Lent, 1983), [4] (Ribeiro-Carvalho et al., 2006), [5] (Silver et al., 1982), [6] (Windrem et al., 1988a), [7] (Smith et al., 1986), [8] (Lefkowitz et al., 1991), [9] (Aughton et al., 1990), [10] (Barth et al., 1984), [11] (Curnes et al., 1986), [12] (Guimiot et al., 2009), [13] (Mehta and Hartnoll, 2001), [14] (Pfizer and Das, 2000), [15] (Revanna et al., 2018), [16] (Silver and Ogawa, 1983), [17] (Smith et al., 1987), [18] (Truwit et al., 1990), [19] (Young et al., 1992).

In our review of the literature, we found that in 12% (11/90) of cases of PBs in humans, there is evidence that a structural cause may constitute the primary etiology. The most common causes of structural disruption in human cases included midline tumors or cysts (Table 3 and Supplementary Table S6), with frequent reports of interhemispheric cysts (7/11) and lipomas (3/11). Interhemispheric cysts are cystic collections in the interhemispheric fissure that may communicate with the ventricular system (Barkovich et al., 2001), whereas midline lipomas are abnormally differentiated meninx primitive that form lipomatous tissue instead of the meninges. Midline lipomas form within the intradural space and occur in the pericallosal region in the interhemispheric fissure approximately 50% of the time. Additionally, there was one report of a midline hamartoma, a benign mass of disorganized tissue, leading to PB development.

### 3.2. Genetic etiologies of Probst bundles

Genetic factors were the most common identified cause of ACC with PB, compromising 83% (75/90) of human cases. Whether specific genetic etiologies lead to ACC with PBs compared to ACC without PBs has remained largely unknown. To investigate influences on PB development, we compiled a list of genes that when independently decreased in expression lead to PB formation, or that have been explicitly reported to not result in PB formation despite producing ACC in mice and humans (Supplementary Table S7). An intriguing hypothesis in the field is that disruption of midline territories is the primary etiology of ACC with PB formation (see section 2.1) (Wahlsten et al., 2006; Gobius et al., 2016). Indeed, a study reviewing human MRIs with ACC (without clear structural etiologies) found that 100% of cases had disrupted midline territories (Gobius et al., 2016). Therefore, we reviewed whether the genes reported to give rise to PBs when misexpressed might be primarily involved in development of midline territories, or another common cause.

**Figure 5:**
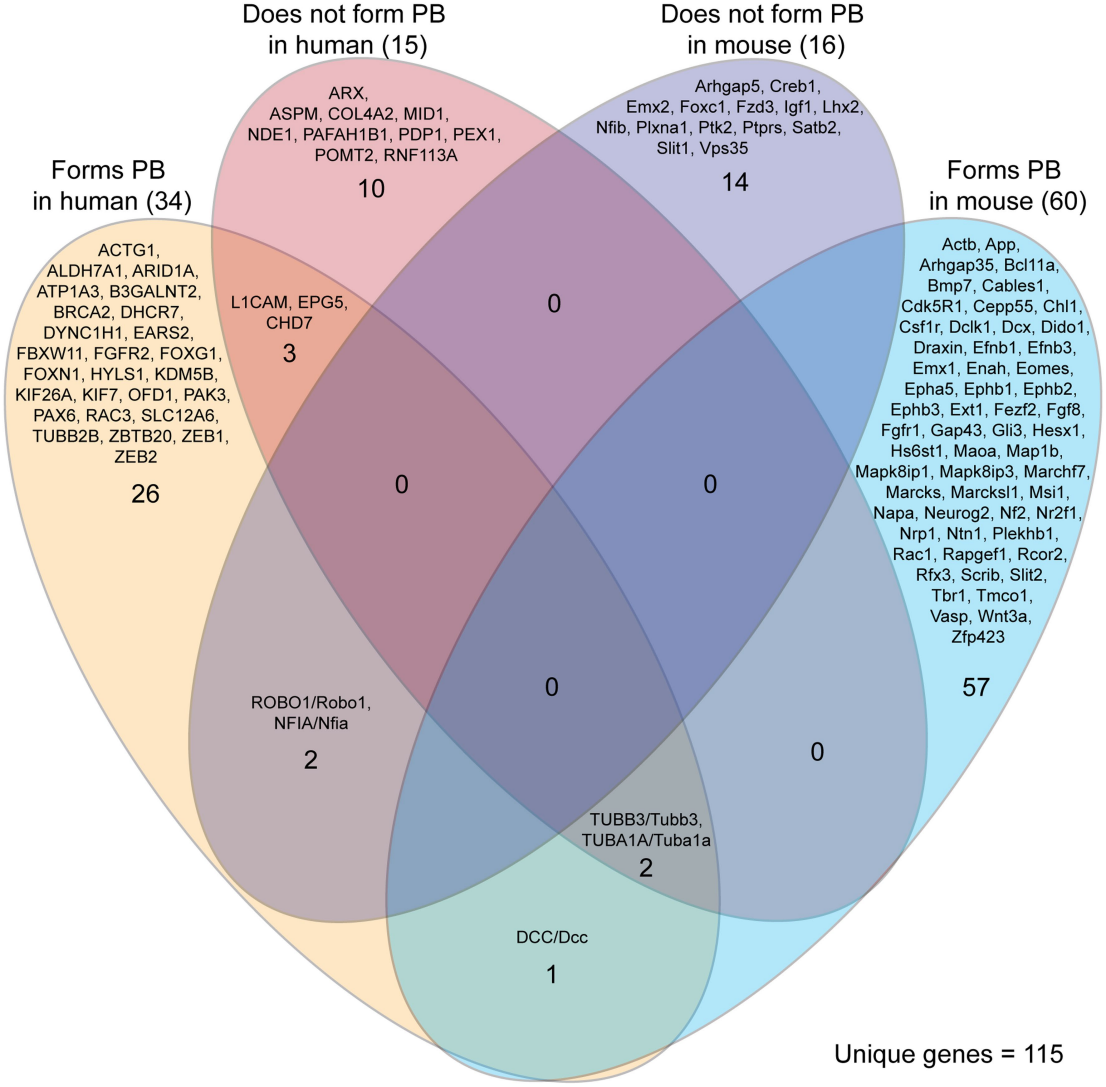
Genes implicated in Probst bundle (PB) formation in mouse and human. Of 235 reports of ACC specifying the presence or absence of Probst bundles, there were 292 cases of genetic etiology implicated in PB formation in mouse and human. Of these 292 cases, there were 115 unique genes identified that, if they are decreased in expression or knocked down, may or may not lead to PB formation. There was little overlap of the same genes being involved in both mouse and human, with three genes (DCC/Dcc, TUBB3/Tubb3, and TUBA1A/Tuba1a) reported that lead to the formation of PB in both mouse and human. Five genes were reported with implications in both PB formation and PB absence in humans (L1CAM, EPG5, CHD7, TUBB3, TUBA1A), reflecting the complexity of correlating genetic data with the formation of PBs.

Our literature search identified 115 unique genes in ACC humans and mice that did or did not lead to PB formation (Figure 5). Most of the genes identified were reported to lead to the formation of PBs (91/115, 79%), with the remainder explicitly reporting an absence of PBs. These proportions may be influenced by a higher tendency to report on the presence of PBs than their absence. Mouse and human genetic reports differed in their experiment type, with mouse reports being more likely to be single gene knock-out experiments, and with human reports often conducting retrospective genetic analyses on single or multiple cases after the phenotype has been revealed. Due to this difference in study types, the genes identified in mouse studies are more likely to directly underlie the PB phenotype. Indeed, this complexity is demonstrated by reports of genes that are associated with both PB formation and PB absence in ACC humans (5 genes in total, Figure 5). This could be due to other polygenic factors that influence gene expression and biological processes and/or the identification of genetic mutations that may not be involved in PB development, but can often be implicated in ACC.

We used the Database for Annotation, Visualization and Integrated Discovery (DAVID) to determine whether human or mouse genes associated with PB formation shared common ontologies, such as the location (cellular component) where the gene product commonly acts. These unbiased analyses of human and mouse genes in DAVID did not reveal any striking patterns in their cellular component ontologies in either species (Tables 4, 5). Instead, we identified a broad diversity of cellular localizations, such as membrane, nuclear, cytoplasm, cytoskeleton, cell projection and cell membrane, indicating that no specific subcellular process may underlie PB formation. Moreover, whether the variety of subcellular localization of causative genes contribute to the disruption of a single cell population, or instead multiple different cell populations remains unclear.

**Table 4:** List of genes implicated in ACC with PBs in mice, organized by cellular component of gene.

	Genes involved (HGNC Symbol)	
Cellular Component	Manipulation produces ACC with PBs	Manipulation produces ACC without PBs
Membrane	30 genes: App, Arhgap35, Cdk5r1, Chl1, Csf1r, Dcc, Efnb1, Efnb3, Epha5, Ephb1, Ephb2, Ephb3, Ext1, Fgfr1, Gap43, Gli3, Hs6st1, Maoa, Mapk8ip1, Marcks, Marcksl1, Msi1, Napa, Nf2, Nrp1, Plekhb1, Rac1, Scrib, Tmco1, Vasp	8 genes: Arhgap5, Creb1, Fzd3, Plxna1, Ptk2, Ptprs, Robo1, Vps35
Cytoplasm	29 genes: Actb, App, Arhgap35, Bcl11a, Cables1, Cdk5r1, Cep55, Dclk1, Dcx, Dido1, Enah, Fgfr1, Gap43, Gli3, Map1b, Mapk8ip1, Mapk8ip3, Marcks, Marcksl1, Msi1, Nf2, Nrp1, Ntn1, Plekhb1, Rac1, Scrib, Tuba1a, Tubb3, Vasp	3 genes: Arhgap5, Ptk2, Vps35
Nucleus	24 genes: Actb, App, Arhgap35, Bcl11a, Cables1, Cdk5r1, Dido1, Efnb1, Emx1, Eomes, Fezf2, Fgfr1, Gli3, Hesx1, Mapk8ip1, Msi1, Neurog2, Nf2, Nr2f1, Rac1, Rcor2, Rfx3, Tbr1, Zfp423	8 genes: Creb1, Emx2, Foxc1, Lhx2, Nfia, Nfib, Ptk2, Satb2
Cell projection	20 genes: App, Arhgap35, Cables1, Cdk5r1, Dclk1, Dcx, Enah, Epha5, Ephb1, Ephb2, Ephb3, Gap43, Gli3, Map1b, Mapk8ip3, Nf2, Rac1, Scrib, Tubb3, Vasp	3 genes: Ptk2, Ptprs, Robo1
Cell membrane	19 genes: App, Arhgap35, Cdk5r1, Chl1, Csf1r, Efnb1, Epha5, Ephb1, Ephb2, Ephb3, Fgfr1, Gap43, Marcksl1, Napa, Nf2, Nrp1, Rac1, Scrib, Vasp	6 genes: Arhgap5, Fzd3, Plxna1, Ptk2, Ptprs, Robo1
Cytoskeleton	12 genes: Actb, Arhgap35, Cep55, Dido1, Enah, Map1b, Marcks, Marcksl1, Nf2, Tuba1a, Tubb3, Vasp	1 gene: Ptk2
Secreted	8 genes: App, Bmp7, Chl1, Draxin, Fgf8, Ntn1, Slit2, Wnt3a	2 genes: Igf1, Slit1
Synapse	5 genes: Enah, Gap43, Map1b, Rac1, Scrib	1 gene: Ptprs

Does not include overexpression studies or where multiple genes were manipulated. Reduced gene expression only. Only listed cellular component categories where at least five genes are listed.

**Table 5:** List of genes implicated in ACC with PBs in humans, organized by cellular component of gene.

	Genes involved (HGNC symbol)	
Cellular component	Manipulation produces ACC with PBs	Manipulation produces ACC without PBs
Cytoplasm	15 genes: ACTG1, ALDH7A1, BRCA2, DYNC1H1, EPG5, FBXW11, HYLS1, KIF26A, KIF7, OFD1, PAK3, RAC3, TUBA1A, TUBB2B, TUBB3	8 genes: ASPM, EPG5, MID1, NDE1, PAFAH1B1, PEX1, TUBA1A, TUBB3
Nucleus	14 genes: ALDH7A1, ARID1A, BRCA2, CHD7, FBXW11, FOXG1, FOXN1, KDM5B, NFIA, OFD1, PAX6, ZBTB20, ZEB1, ZEB2	5 genes: ARX, ASPM, CHD7, PAFAH1B1, RNF113A
Cytoskeleton	11 genes: ACTG1, BRCA2, DYNC1H1, HYLS1, KIF26A, KIF7, OFD1, RAC3, TUBA1A, TUBB2B, TUBB3	6 genes: ASPM, MID1, NDE1, PAFAH1B1, TUBA1A, TUBB3
Membrane	11 genes: ATP1A3, B3GALNT2, BRCA2, DCC, DHCR7, FGFR2, OFD1, PAX6, RAC3, ROBO1, SLC12A6	5 genes: L1CAM, MID1, PAFAH1B1, PEX1, POMT2
Cell projection	7 genes: HYLS1, KIF7, L1CAM, OFD1, RAC3, ROBO1, TUBB3	2 genes: L1CAM, TUBB3
Cell membrane	5 genes: ATP1A3, FGFR2, RAC3, ROBO1, SLC12A6	1 gene: L1CAM
Microtubule	5 genes: DYNC1H1, KIF26A, TUBA1A, TUBB2B, TUBB3	5 genes: MID1, NDE1, PAFAH1B1, TUBA1A, TUBB3

Only listed cellular component categories where at least five genes are listed.

Several genes associated with ACC and PBs have been directly evidenced to act at the midline, with their misexpression associated with failure to establish a permissive substrate for callosal axon crossing. Fgf8 has been demonstrated to be an important effector in this process, triggering an astrogliogenic program of tissue remodeling at the midline, with transcription factors Nfia and Nfib acting upstream in this pathway. Directed site-specific electroporation of Fgf8 into the midline territory itself in WT, Nfia, and Nfib mutant mouse models, as well as Fgf8 mutant mouse models, revealed that precise and timely expression of Fgf8 is required for midline remodeling, and that disruptions to this process invariantly lead to the formation of AC with PBs (Gobius et al., 2016).

In addition to the gene disruptions evidenced to primarily disrupt midline territories, individual gene defects may also affect multiple processes in brain development. For example, many gene defects reported in mice to cause ACC with PB have well-established roles in cortical neuron development, however recently some of these have also been shown to have additional roles in midline glia remodeling (Morcom et al., 2021a,b). An example of this is one of the many axon guidance systems involved in callosal formation: the secreted protein DRAXIN and its axon guidance receptor Dcc. Callosal axons express DCC during midline crossing, which interacts with DRAXIN expressed at the midline and contributes to the guidance of callosal axons across the midline (Ahmed and Shinmyo, 2021). However, DCC and DRAXIN also have a direct effect on midline remodeling, which precedes their roles in axon guidance. Mouse models with Dcc mutations display impaired midline remodeling, with defects in morphology, distribution, and migration of midline zipper glia along the midline (Morcom et al., 2021b). In addition, inbred BTBR/C57 mice, which have a truncated DRAXIN protein as well as other mutations, have defects in midline zipper glia migration and proliferation (Morcom et al., 2021a). Whether many other, or perhaps even all, gene misexpressions that result in PBs similarly have roles in midline remodeling is an interesting question for future research and will require precise spatiotemporal experiments to specifically misexpress genes either in cortical axons or the midline in isolation of one another (Table 6).

**Table 6:** Additional non-structural and non-genetic causes of ACC that lead to PB development in mouse and humans.

Type of defect	Number of cases		Citations
	Mouse	Human	
Environmental (Gamma irradiation)	2	0	3, 5
Infectious (Zika virus)	0	1	4
Gross neurodevelopmental malformations	0	3	1, 2, 6

Citation key: [1] (Tavano et al., 2022), [2] (Hannay et al., 2009), [3] (Abreu-Villaça et al., 2002), [4] (Cachay et al., 2020), [5] (Caparelli-Dáquer and Schmidt, 1999), [6] (Wang et al., 2022).

The hypothesis that genetic causes of ACC with PB are primarily due to changes in midline remodeling introduces the question of whether interventions targeting the midline in early development may be used as a potential treatment option. A glial-coated midline implant was reported to restore some midline crossing in mouse brains with ACC from a structural cause (Silver and Ogawa, 1983), however this has never been replicated and it is unknown whether various genetic or other structural causes of ACC with PB could also be restored with a callosal bridge. Future experiments with attempts to “rescue” the ACC with PB phenotype with a glial coated bridge or other midline intervention are necessary to further probe this as a potential therapy.

### 3.3. Other etiologies of Probst bundles

In addition to structural and genetic causes of ACC with PBs, there are also reports of infectious and environmental causes. We identified reports in mouse of gamma irradiation and a report in human fetus of Zika virus infection leading to ACC with PB formation (Supplementary Table S8). Gamma irradiation is thought to produce ACC with PBs via a failure of midline remodeling due to an absence of midline glia (Abreu-Villaça et al., 2002). Possible mechanisms for ACC with PB development in the case report of Zika virus infection in fetal human (Cachay et al., 2020) includes disruption of midline glial cells (Li et al., 2018), decreased vasculature limiting cortical growth (Garcez et al., 2018) and a recent preprint reporting a reduction of proliferating cortical cells, intermediate progenitors and SATB2+ neurons (Christoff et al., 2021). It remains unclear whether viral-mediated neurodevelopmental deficits generally have the potential to produce ACC with PBs, perhaps when structural disorganization is less extreme.

### 3.4. Agenesis of the corpus callosum without Probst bundle formation

To understand the conditions sufficient and required for PB formation, we also reviewed cases of callosal agenesis in which PBs do not form. There were 39 reports in humans and 30 reports in mice in which ACC was concomitant with an absence of PBs (Supplementary Table S5). The majority of these cases of ACC without PBs involved major nervous system malformations (Hetts et al., 2006), such as meningiomyelocele/Chiari II malformations (Loeser and Alvord, 1968; Özek et al., 2008; Sundarakumar et al., 2015), or classic holoprosencephaly (Magee and Olson, 1961). The mechanisms implicated in these cases include deficits deficits in genes encoding growth factors (e.g., Ifg1), various tubulins and proteins associated with cellular metabolism (e.g., Pdh), suggesting that broad deficiencies in growth, axonal outgrowth and metabolism may contribute to the gross disorganization of ACC brains without PBs (Supplementary Table S5). However, there are three case reports of such gross neurodevelopmental malformations that produce ACC concomitant with PBs, including syntelecephaly, myelomeningiocele, and Chiari II malformations, demonstrating that it is possible for PBs to form in these conditions (Hannay et al., 2009; Tavano et al., 2022; Wang et al., 2022). The presence of PBs in ACC may therefore signify a more complex and organized brain overall, and future studies systematically associating the degree of structural disorganization with the presence or absence of PBs would help to inform this relationship (Hetts et al., 2006).

Cumulatively, this evidence suggests that PBs form in almost all cases of callosal agenesis where the structure of the brain, and particularly that of the cortex, is not significantly distorted. A few rare exceptions to this are reports of genetic mouse models of ACC where instead of forming PBs, the axons appear halted on either side of the midline, such as in Slit2 KO, Robo1 KO, or Satb2 KO mice (Bagri et al., 2002; Andrews et al., 2006; López-Bendito et al., 2007; Alcamo et al., 2008; Britanova et al., 2008; Unni et al., 2012; Magnani et al., 2014; Supplementary Table S2). However, as many of these mouse models are perinatally lethal, it remains unknown whether axon growth is simply delayed and these axons would have eventually formed PBs postnatally. Perhaps the most likely of these to be a true example of callosal absence/malformation without accompanying PBs is the Satb2 knockout, as a conditional Emx1-Cre Satb2 flox/flox mouse model displays a hypoplastic callosal phenotype without obvious PBs postnatally. Interestingly, this mouse model displays non-crossing axons aberrantly projecting ventrally in a fashion similar to the septal projections of PBs (described in section 2.3), indicating that this feature may be developmentally distinct from PBs (Leone et al., 2015). A possible reason why PBs are not apparent in Satb2 KO animals is because would-be callosal neurons do not have repression of CTIP2 by SATB2, and SATB2 is usually expressed in mouse cortical neurons after E12.5, corresponding to the initiation of a switch from lateral to medial projection direction (Hatanaka et al., 2016). Therefore, cortical axons of Satb2 KO animals may never undergo a switch from lateral to medial projection fate, leading to neurons committing to alternative lateral pathways including the corticofugal tract or the anterior commissure (Alcamo et al., 2008; Britanova et al., 2008). It is therefore possible that this particular manipulation respecifies callosally-projecting neurons to other projection fates, and therefore there are insufficient axons arriving at the midline to form PBs. Re-routing through the anterior commissure is of particular interest as monotremes and marsupials do not have a corpus callosum and use instead the anterior commissure as their primary interhemispheric tract, providing the intriguing hypothesis that brains have potential to use evolutionarily older projection pathways as a plasticity mechanism (Fenlon et al., 2021). Re-routing of axons through a separate, non-callosal interhemispheric tract has also been reported as a possibility in a DTI study in acallosal humans (Tovar-Moll et al., 2014). Understanding the mechanisms underlying the formation of ectopic bundles versus re-routing through existing commissures and tracts remains a central question for understanding the preservation of interhemispheric communication in many of these phenotypes.

## 4. Probst bundle function and behavioral/cognitive significance

Despite constituting the largest stereotypical ectopic brain tract in humans, it remains unclear whether PBs are functional during development and/or in the adult, and whether this functionality is beneficial, neutral, or detrimental to cognitive outcomes. In addition to the lack of axonal elimination during development that may point to PBs making functional connections (see section 2.1), they also have features of mature brain structures, including myelination patterns that are similar to a neurotypical corpus callosum (Müller et al., 1996; Smith et al., 1996; Demyanenko et al., 1999; Magara et al., 1999; Meixner et al., 2000; Kelkar et al., 2003; Bénézit et al., 2015; Sforazzini et al., 2016). Indirect evidence indicating that PBs may be functional have included studies employing glucose uptake synchronization (Magara et al., 1998), EEG coherence (Lazarev et al., 2016), fetal connectome (Jakab et al., 2015), and electrophysiology (Lefkowitz et al., 1991). These have collectively reported either an increase in ipsilateral anteroposterior connectivity in acallosal brains with PBs, or additional evidence of neural activity, like electrophysiological field measurements in the vicinity of PBs and glucose uptake in regions along the PB. All reports have only correlated PB presence with functional activity, therefore future experiments directly and specifically manipulating PBs and measuring resulting brain activity are necessary to definitively characterize their electrical and/or synaptic activity.

If PBs are functional, the question remains whether this function is compensatory, neutral, or maladaptive to cognitive outcome. The presence of PBs is associated with a better neurodevelopmental outcome and performance on behavioral tasks in ACC human studies (Jeeves et al., 2001; Al-Hashim et al., 2016). However, it is difficult to compare ACC cases of PB presence and absence, as brains are more likely to have gross malformations in other regions when PB are absent (see section 3.4). To bypass these inherent differences, instead of comparing ACC brains with and without PB, it may be informative for future studies to assess functional outcome in brains with varying amounts of PB development in the complete to partial ACC spectrum.

It might be expected that cases of partial ACC have better behavioral outcomes than complete ACC (regardless of PB presence/absence) due to less drastic changes in brain anatomy. However, there is evidence that the contrary may be the case. A connectome study found that humans with complete ACC maintain similar functional connectivity patterns as controls. In contrast, in callosal hypoplasia, there were abnormal structural and functional connectivity patterns relative to healthy controls (Szczupak et al., 2021). This may relate to general disorganization of cortical projection neurons inconsistently projecting into the ectopic PB versus through the callosal remnant. This is highlighted by a DTI/HARDI study reporting that homotopic connections do not necessarily correlate with the position or size of the residual corpus callosum, resulting in high variability in connectivity patterns in partial ACC compared to complete ACC (Wahl et al., 2009; de Carvalho Rangel et al., 2011). This high variability of connectivity patterns could result in greater variation of behavioral and cognitive performance (de Carvalho Rangel et al., 2011), potentially leading to both worse and better outcomes when compared with the average outcome of complete ACC. Another potential explanation is provided by a recent report suggesting that partial ACC is more frequently concomitant with other brain abnormalities than complete ACC (Li and Wang, 2021), however, more studies are required to confirm this relationship.

While it is unknown whether PBs are behaviorally significant, their presence in ACC correlates with frequent behavioral phenotypes, such as maintenance of interhemispheric communication and autistic behaviors. ACC individuals (PB status not consistently reported) demonstrate preservation of interhemispheric connectivity on behavioral and resting-state functional magnetic resonance imaging studies (Jeeves, 1969; Paul et al., 2007; Tyszka et al., 2011; Owen et al., 2013; Roland et al., 2017), and evidence from virtual lesions of human PBs on connectome imaging support that PBs may be involved in interhemispheric communication (Owen et al., 2013). This contrasts with cases of callosal surgical separation later in life, i.e., callosotomy, where a disconnection, or “split-brain,” syndrome characterized by mild to severe neuropsychological symptoms can result (Sperry, 1961). Individuals born with ACC, and children who receive callosotomy early in life, frequently do not display the disconnection syndrome (Saul and Sperry, 1968; Ptito and Lepore, 1983; Lassonde et al., 1986, 1988, 1991). Whether PBs contribute to the maintenance of interhemispheric communication is unclear. Some potential mechanisms that would enable PBs to maintain interhemispheric communication include a direct anatomical connection, for example via the hippocampal commissure or subcortical routes (see section 2.3), or strengthened ipsilateral connectivity with cortical hubs may contribute to novel polysynaptic interhemispheric communication methods that utilize other pre-existing interhemispheric circuits.

In addition to maintained interhemispheric communication, PBs are also highly associated with “syndromic” diagnoses, most commonly autism spectrum disorder (Edwards et al., 2014, Paul et al., 2014). Autism is a neurodevelopmental disorder of miswired brain connections that commonly involves an altered structural and functional connectivity of the corpus callosum, including reduced callosal volume (Valenti et al., 2020) and an associated decrease in interhemispheric connectivity (Yao et al., 2021). Understanding the potential developmental plasticity of brain connections, such as via ectopic tracts like PB, may help us better understand how neurodevelopmental conditions of miswiring occur, as well as the rules and limitations of compensatory plasticity.

## 5. Discussion

Roger Sperry reported that adult humans who had undergone a callosotomy commonly displayed a “disconnection syndrome,” involving disrupted communication between the left and right sides of the body (Sperry, 1968). Despite early observations that people who never develop a corpus callosum do not display disconnection syndrome, the morphological substrates facilitating intact interhemispheric communication in those cases have remained unclear. Here, we review the current understanding of the development, anatomy, etiology and functionality of PBs, the largest ectopic axon tract to predictably form under any known condition in the brain of placental mammals, and which exclusively form in cases where the corpus callosum is wholly or partially absent developmentally. While this bundle has been described as formed by tangled and perhaps dysfunctional axons, its persistence into adulthood, topographic arrangements and patterns of connectivity suggest that it may have functional roles. Although many structural and genetic etiologies underlie PB formation, their broad anatomy has a remarkable consistency both within and between species. This consistency, as well as correlations between PB presence and overall function suggest that these connections may provide cognitive and/or behavioral compensation in preserving interhemispheric communication in callosal absence.

A renewed focus on naturally and predictably occurring ectopic axon tracts may help us to better understand the rules and limitations of axon plasticity, as well as the mechanisms underlying developmental disorders of miswiring. It remains unclear whether encouragement or discouragement of axon plasticity in diverse developmental connectivity disorders might improve or worsen cognitive outcome, however the possibility of harnessing these mechanisms for candidate therapies is an intriguing area for future investigation. Further understanding of the cognitive and behavioral significance of PBs may therefore extend to other injuries/developmental malformations of the brain to help answer the broader question of why development offers a greater capacity for functional plasticity, and which mechanisms could be harnessed in the adult to aid cognitive outcome.

## Author contributions

LF: Conceptualization, Funding acquisition, Investigation, Supervision, Writing – review & editing. ZL: Conceptualization, Investigation, Writing – original draft, Writing – review & editing. RS: Funding acquisition, Investigation, Supervision, Writing – review & editing.
